# St. Mark's Hospital and the Prince of Wales's Fund

**Published:** 1900-03-10

**Authors:** 


### The Institutional Workshop.

### ST. MARK'S HOSPITAL AND THE PRINCE OF WALES'S FUND.

Sir Savile Crossley writes as follows in reply to Mr. Martin: "Though, like Mr. R. B. Martin, no professional accountant, I have the benefit of the advice of a chartered accountant, and my statement of the accounts of this hospital was made after consultation with him. In his anxiety to attack the fund Mr. Martin has impaled himself on one of the horns of a dilemma. The published accounts in question, those for 1898, duly certified by the professional auditors, contain the following: 'Total ordinary income, £4,986 8s. 4d.; total ordinary expenditure, £3,429 18s. 5d.' This obviously shows a surplus of £1,556 9s. 11d. Mr. Martin, the treasurer, now writes saying that there was no surplus, but 'for that year a deficiency of £1,158.' Either Mr. Martin is correct in this statement, in which case he as treasurer has published incorrect accounts; or if the published accounts of the hospital are correct, then his letter is not. In either case it is unreasonable to blame the committee of the Prince of Wales's Fund for relying on the printed and audited accounts of the hospital. I regret that an authority of the hospital should have initiated this discussion in the public Press, but trust it may result in raising the financial management of the hospital to the level of its medical standard."

Mr. T. Mark Merriman, of 25, Austin-friars, E.C., writing to the Press on February 27th, says: "As the originator of the resolution which has been the cause of the recent correspondence under the above heading, may I be permitted to say that the accounts of St. Mark's Hospital are made out on the lines prescribed by the committees of the Hospital Sunday and Saturday Funds respectively? It is to be regretted that in the arrangement of the few items of income, in all ten in number, the item 'donations' did not show that it comprised special sums of, amongst others, £1,025, which should clearly be capitalised; but the fact that £1,720 8s. 6d., part of the festival dinner fund, and the three items comprising the contributions from the Hospital Sunday and Saturday Funds and the Prince of Wales's Fund, all voted in 1897, though brought into the 1898 accounts, and amounting altogether to £158 3s., should surely show at a glance, to anyone accustomed to such accounts, that there was a deficiency of ordinary income of at least £1,551, or nearly one-half of the expenditure. Mr. Martin's letter of the 17th inst. shows that, as a matter of fact, the ordinary income is very insufficient for the needs of the hospital, and it is to be hoped that now the committee of the Prince of Wales's Fund have been made aware of the true facts of the case, they will do what is right by St. Mark's Hospital, as, after all, they are only stewards of money given by the public for hospitals generally, in the full reliance that the name of the First Gentleman in the Land shall be a guarantee of fair distribution; and they, therefore, are not entitled to favour one hospital more than another, where the need of funds can be shown."

---

Sir,—I again beg your indulgence in order to reply to two points raised in Mr. Merriman's letter.

In it he confesses that St. Mark's Hospital accounts were published in a manner unsatisfactory to some of the hospital authorities, simply because they "are made out on the lines prescribed by the committees of the Hospital Sunday and Saturday Funds respectively."

This admission is a well-deserved compliment to both funds mentioned, and proves the advantage of central funds, which place before the public the correct financial condition of the hospitals.

Further than this, it shows that the Prince of Wales's Fund, in accepting the figures already accepted by the Sunday and Saturday Funds, has incurred no new responsibility.

Your correspondent also says, "But the fact that £1,720 8s. 6d., part of the festival dinner fund, and the three items comprising the contributions from the Hospital Sunday and Saturday Funds and the Prince of Wales's Fund, all voted in 1897, though brought into the 1898 accounts, and amounting altogether to £158 3s., should surely show at a glance, to anyone accustomed to such accounts, that there was a deficiency of ordinary income of at least £1,551, or nearly one-half of the expenditure."

In this complex sentence Mr. Merriman, regardless of grammar, relies on a "fact" which he never reaches. If there is any meaning in this statement, it is that no part of the festival dinner or of the grants from the three funds should be treated as income, but that all these sums should be capitalized.

As regards the festival dinner fund, if this view is correct, Mr. Merriman censures his own committee for classifying a part of these proceeds as ordinary income. Surely such criticisms would be better made privately in committee than in the Press.

As regards the grants from the three funds, we have yet to learn that any of these grants are intended to be treated as capital by their recipients.

And if your correspondent's views were adopted, I venture to assert that the subscribers to these funds would withdraw their support.

It is gratifying to all interested in the central funds to find statistics arrived at by each confirmed by the other two, as in the present instance.—Your obedient servant,

Savile Crossley.

12, Carlton House Terrace, S.W., March 5th.

